# Between Risk and Refuge: Anthropogenic Linear Features Serve as Barriers, Corridors, and Habitat for Eastern Copperheads (*Agkistrodon contortrix*)

**DOI:** 10.1002/ece3.73471

**Published:** 2026-04-13

**Authors:** Ethan J. Kessler, Shelly N. Colatskie, Brittany I. Neier, Benjamin C. Jellen

**Affiliations:** ^1^ Illinois Natural History Survey, Prairie Research Institute University of Illinois at Urbana‐Champaign Champaign Illinois USA; ^2^ National Great Rivers Research and Education Center Lewis and Clark Community College East Alton Illinois USA; ^3^ Missouri Department of Conservation Jefferson City Missouri USA; ^4^ Independent Researcher St. Louis Missouri USA; ^5^ Basic Sciences Department University of Health Sciences and Pharmacy St. Louis Missouri USA

**Keywords:** habitat selection, radio telemetry, random steps, road ecology, snake, step selection function, wildlife movement

## Abstract

Urbanization creates vast human‐altered landscapes effectively isolating remaining pockets of habitat and introducing linear anthropogenic features such as roads and utility corridors. These features may function as barriers, corridors, or habitat, potentially in unexpected ways. We investigated the spatial ecology of eastern copperheads ( *Agkistrodon contortrix* ) in a suburban nature park in the St. Louis metropolitan area (Missouri, USA) to determine the extent that roads, paved foot trails, and powerline clear cuts influence movement and habitat selection. Using radiotelemetry, we tracked 14 adult snakes (7 males and 7 females) over six active seasons (2018–2023), collecting 1950 relocations. Random path analyses, step selection functions, and context‐dependent movement models uncovered an apparent paradox; snakes of both sexes avoided crossing roads at all spatial scales, yet preferred roadside habitat. Both sexes also selected for foot trail crossings and occupancy of powerline clear cuts, potentially utilizing these features for improved foraging habitat, thermoregulatory opportunities, and refugia. Additionally, males moved further per step than females, but there was no difference in movement probability between the sexes. Snakes' odds of moving were 46% lower when occupying roadside habitat and 2.3 times higher when occupying powerline clear cuts, suggesting differing behavioral states within, and functions of, these habitats. Our findings suggest that linear anthropogenic features can simultaneously act as barriers, movement corridors, and preferred habitat, underscoring the need to determine whether such features provide genuine benefits or function as ecological traps for wildlife occupying urban landscapes.

## Graphical abstract

Urbanization introduces linear anthropogenic features which have potential to hinder or facilitate wildlife movement. Using 6 years of radiotelemetry (1950 relocations) on a population of eastern copperheads in an urban nature park, we found snakes consistently avoided road crossings, yet selected for roadside habitat, trail crossings, and powerline clear cuts. These results show the potential for linear anthropogenic features to act as both risks and resources, underscoring the importance of distinguishing actual beneficial habitat from ecological traps in fragmented landscapes.

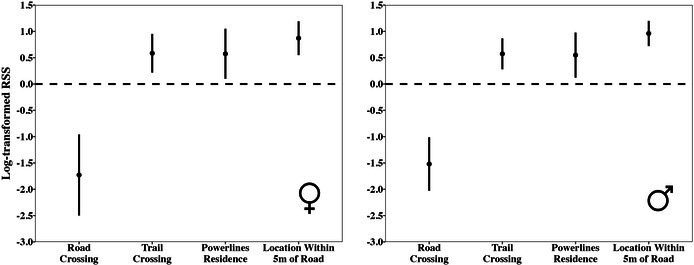

## 1 Introduction

Human alteration of natural ecosystems is a primary driver of habitat loss and decreasing biodiversity (Jaureguiberry et al. 2022). Urbanization in particular is a pervasive global force (Haddad et al. 2015) resulting in habitat fragmentation in urban and suburban areas (Radeloff et al. 2005; Bozkurt and Basaraner 2024). Large‐scale land conversion has resulted in widespread habitat loss often leaving small, isolated patches of suitable wildlife habitat which are further impacted by human infrastructure (e.g., roads and utility corridors) further bisecting habitat (Torres et al. 2016). The presence of these and other anthropogenic landscape features can reduce population viability through increased mortality (i.e., roadkill) and/or reduced connectivity to surrounding populations (Bonnet et al. 1999; Coffin 2007). Such linear features can also reduce suitable habitat by creating corridors of edge habitat and reducing the amount of contiguous interior habitat. Therefore, as urbanization and land conversion destroy and isolate habitat, it is important to understand the cumulative effects that these forces have on wildlife populations.

One particularly acute impact of anthropogenic landscape features on wildlife populations is altered movement and habitat selection. Linear anthropogenic features can simultaneously function both as barriers and as corridors for wildlife movement. Roads are one of the most significant barriers to wildlife movement, particularly for small‐bodied terrestrial species, both through direct mortality via vehicle strikes and through road avoidance behavior limiting dispersal and population connectivity (Andrews and Gibbons 2005; Fahrig and Rytwinski 2009; Holderegger and Di Giulio 2010), which can ultimately decrease gene flow between subpopulations (Shepard et al. 2008). For terrestrial snakes, the thermoregulatory benefits of managed roadside habitat provide for gravid female viviparous species (Mccardle and Fontenot 2016; Sisson and Roosenburg 2023) and present an additional ecological trap via road mortality due to their relatively slow escape behavior and their limited perceptual distance of approaching vehicles (Roe et al. 2006; Row et al. 2007). Paved walking trails might provide similar perceptual benefits without the added risk of vehicle‐caused mortality. Additionally, there are potential benefits in novel habitat created by roads via edge effects, altered microhabitat features, and modified vegetation communities through maintained right‐of‐ways (Rhoden et al. 2016; George et al. 2017; Sisson and Roosenburg 2023). Utility corridors, such as powerline clear cuts, provide similar benefits in some cases (Clarke et al. 2006; Latham et al. 2011; Bartzke et al. 2015). Yet, habitat quality can also suffer if these features reduce foraging opportunities and/or refuge sites (Strevens et al. 2008). The complex interplay of positive and negative impacts of linear anthropogenic features is largely contingent on the context of these disturbances and the traits of the impacted wildlife.

Terrestrial snakes, such as the eastern copperhead ( *Agkistrodon contortrix* ), are ideal taxa for examining wildlife responses to anthropogenic features in fragmented habitats. As sit‐and‐wait predators with annual dependence upon fixed hibernacula, *A. contortrix* is sensitive to habitat alteration and has limited emigration capacity when local habitat becomes unsuitable, particularly in the harsh matrix of suburban sprawl (Fitch 1960; Smith et al. 2009). However, this species shows some tolerance for such isolated habitat patches persisting in suburban parks throughout their range, suggesting some degree of adaptation to human‐modified habitats (Gloyd and Conant 1990). Additionally, there is some evidence to suggest that *A. contortrix* , and other temperate snake species, have complex relationships with human‐modified habitats. Many species exhibit substantial road avoidance behavior (Andrews and Gibbons 2005) but also show an affinity for edge habitat (which is often created and maintained along roadsides) for its foraging and thermoregulatory benefits (George et al. 2017; Sisson and Roosenburg 2023). Additionally, many snake species, including *A. contortrix* , exhibit sex‐ and ontogenetic‐specific movement patterns (Jellen and Kowalski 2007; Smith et al. 2009; Sutton et al. 2017; Christensen et al. 2025), which can introduce demographic consequences for differential responses to anthropogenic features. Yet, few studies have examined how the effects of anthropogenic landscape features influence the movement of snakes beyond road avoidance behavior.

Understanding how *A. contortrix* and other wildlife species are affected by anthropogenic landscape features has important implications for how we understand the persistence of wildlife populations in human‐dominated landscapes. For instance, if anthropogenic features serve as barriers to movement, then mitigation efforts should focus on reconnecting isolated subpopulations, while planning for future development should incorporate such impacts. Alternately, some anthropogenic features may confer benefits to wildlife, reducing the need for ameliorating potential impacts in urban and suburban contexts. As land development continues to encroach on remaining habitat, identifying the threats and potential benefits of human infrastructure on wildlife is vital to maintaining viable populations in human‐dominated landscapes.

We used radiotelemetry to evaluate the movements of *A. contortrix* in a suburban nature park in relation to anthropogenic landscape features. Specifically, we used complementary analytical approaches, including random paths analyses and step selection functions, to examine the following objectives at multiple spatiotemporal scales: (1) quantify the responses of *A. contortrix* to linear features including roads, roadside habitat, foot trails, and powerline clear cuts through analyses of crossing behavior and habitat selection; (2) determine if responses to anthropogenic features are sex‐specific; and, (3) evaluate context‐dependent movement behavior to determine if site fidelity varies by habitat type (i.e., roadside right of way or powerline clear cuts).

## 2 Methods

### 2.1 Study Area

**FIGURE 1:**
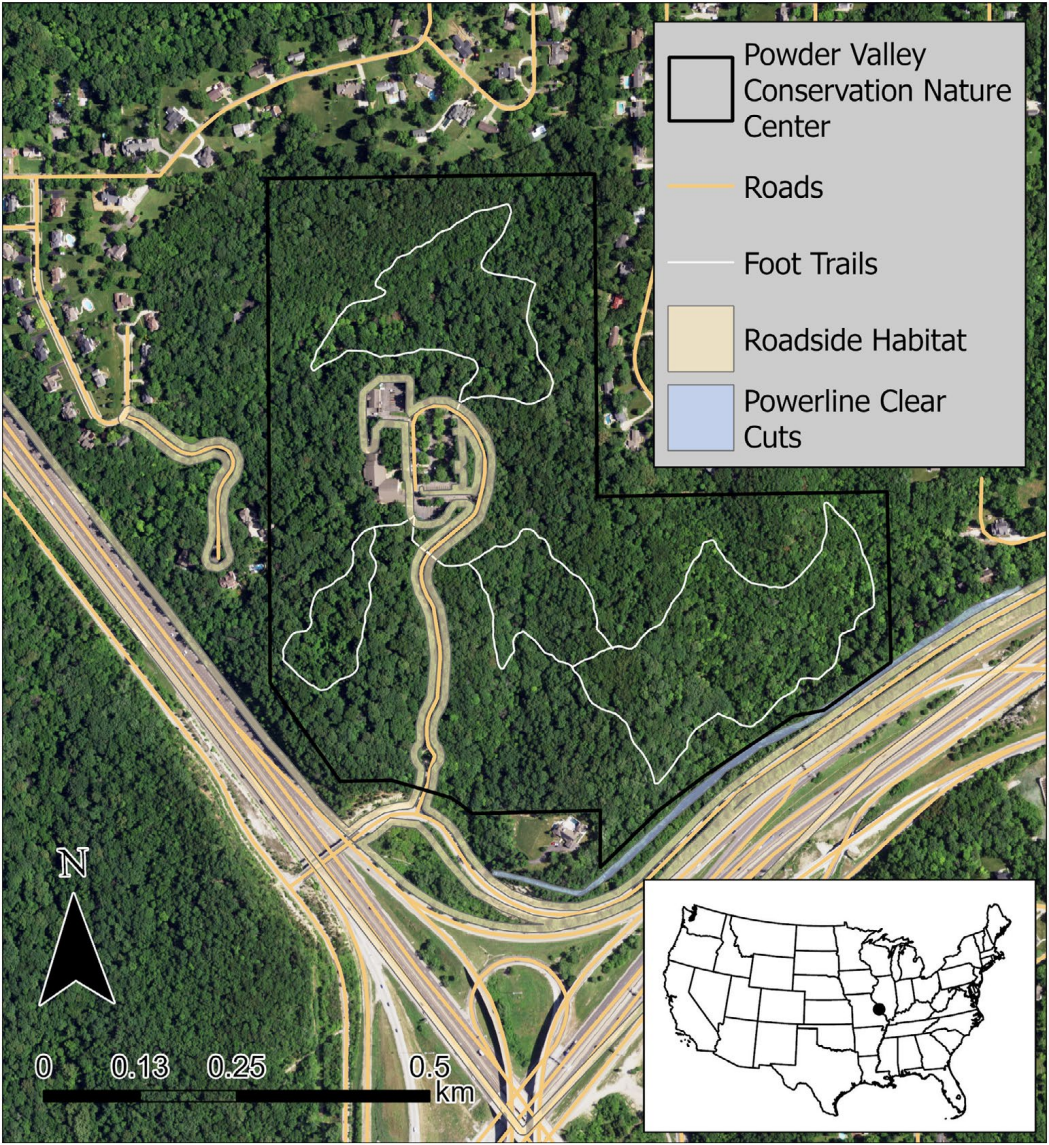
Map of the study area, Powder Valley Conservation Nature Center (black outline), and surrounding area. Anthropogenic linear features are identified in the map including roads (yellow lines), foot trails (white lines), roadside habitat (yellow shaded polygons), and powerline clear cuts (blue shaded polygons).

**FIGURE 2:**
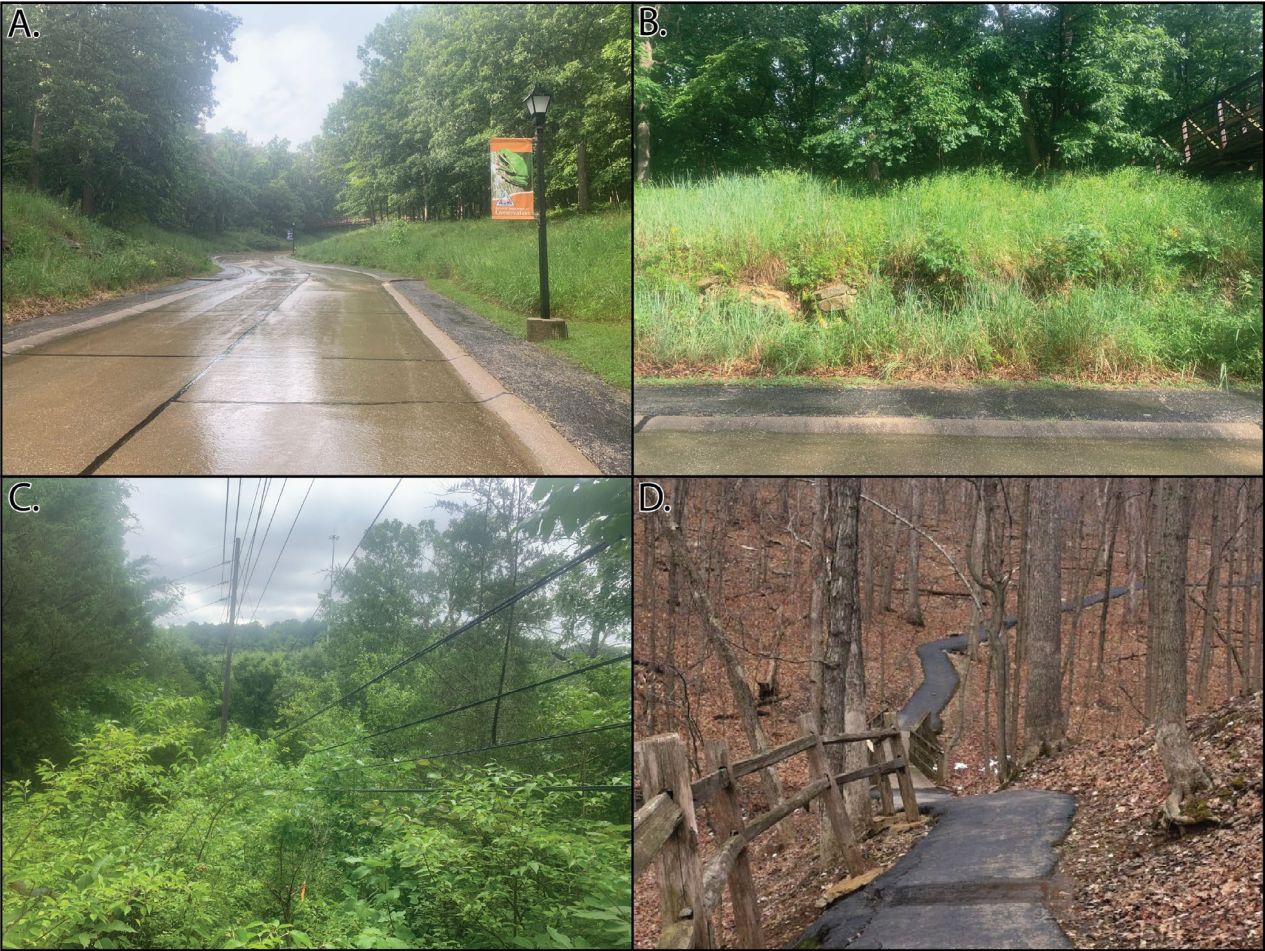
Examples of linear habitat features examined in this study, including (A) central access road for Powder Valley Conservation Nature Center, (B) typical roadside habitat within the park, (C) example of habitat within the powerline clear cut, and (D) example of the paved trails within the park.

This study was conducted from 2018 to 2023 at Powder Valley Conservation Nature Center (PV) in St. Louis County, MO, USA (Figure 1). This Missouri Department of Conservation managed area is situated on 45 ha of oak‐hickory forest in the greater St. Louis metropolitan area and hosts ~80,000 visitors annually. The public grounds are bordered by residential areas to the north and east, and by interstates to the south and west (Figure 1). These features serve to create distinct edges to the natural habitat afforded within the park, and paved trails, an entrance drive, and power line clear cuts further fragment this landscape (Figure 2). The grassy vegetation buffering both sides of the entrance drive is maintained via periodic prescribed burns and affords additional edge habitat.

### 2.2 Field Data Collection

Adult snakes were hand collected opportunistically throughout the active seasons (early April through early November), sexed by cloacal probing (Schaefer 1934), weighed, measured (SVL), and permanently marked by injecting passive integrated transponder (PIT) tags subcutaneously at initial capture. We implanted individuals with SB‐2 T or SI‐2 T; 5.0 or 11.0 g HOLOHIL Systems (Carp, Ontario) radio transmitters at the Animal Hospital of the St. Louis Zoo by staff veterinarians following the guidelines of Reinert and Cundall (1982). Transmitters accounted for less than 6% of the snake's body mass. Following radio implantation, all individuals were released at their respective points of capture.

At each radiotelemetry location, we recorded an initial GPS reading (NAD 83–UTM) and marked it with a uniquely numbered flag to identify unique locations. GPS coordinates were measured using a Garmin GPSMAP 64 s series unit with waypoint averaging. Since *A. contortrix* can make very small movements or remain sedentary between relocations, care must be taken to ensure that error from GPS units does not overestimate movement behavior. Therefore, we followed the following protocol for recording relocations. Movements 1 m or less were not considered unique locations and were assigned identical coordinates. For movements greater than 1 m, we recorded a second GPS reading on a subsequent day under different satellite geometry and averaged the two coordinates if they were within 3 m of one another. If the coordinates were not within 3 m of one another, we took additional readings under different satellite geometries until at least two readings met this requirement. We relocated individuals via radio telemetry an average of 6.9 times weekly during the active season.

### 2.3 Data Preparation

#### 2.3.1 Spatial Data

We developed two anthropogenic linear feature layers for our analysis for roads and foot trails and two polygon features representing powerline clear cuts and a 5 m buffer of roadside habitat within and adjacent to the park. We used a combination of publicly available National Agriculture Imagery Program (NAIP) imagery (U.S. Department of Agriculture 2022; 0.6‐m resolution; collected 2022‐06‐18) and lidar data (Missouri Spatial Data Information Service 2012; collected 2012‐01‐29–2012‐02‐01) to develop the trails and powerlines layers. All imagery and lidar data were processed and interpreted using ArcGIS Pro 3.3.2 (ESRI, Redlands, CA, USA). Powerline clear cuts were interpreted from the NAIP imagery and a tree canopy height layer derived from lidar data. Foot trails were partially obstructed from view due to tree canopy cover in imagery data; therefore, in conjunction with an approximate map of foot trails available for PV, we used lidar data to help interpret these features. To visualize trails, we constructed an intensity of lidar returns layer using only last returns at a 1 m resolution, which made the paved foot trails visible. Roads layers were adapted from the United States Census Bureau's Topologically Integrated Geographic Encoding and Referencing (TIGER) roads layer (U.S. Census Bureau 2022). The buffered roads footprint polygon was created by tracing the outline of the paved roads within and surrounding PV in ArcGIS Pro. We then used the Buffer tool to create a 5 m buffer around this polygon to create a polygon of all habitat within 5 m of a road.

#### 2.3.2 Animal Movement Data

*A. contortrix* are known to exhibit sex‐specific movement patterns (Petersen 1995; Smith et al. 2009; Sutton et al. 2017; Carrasco‐Harris et al. 2020); therefore, we tested for differences in movement frequency and step length using a mixed effects hurdle model with a gamma distribution using the glmmTMB package in R v. 4.3.3 (Brooks et al. 2017). Our model showed no difference in movement frequency, but males exhibited significantly greater mean step lengths (Appendix S1). Therefore, we treated male and female movements separately in all analyses.

#### 2.3.3 Habitat Selection and Feature Crossing

Since we were assessing habitat selection using both line and polygon features, we used movement paths and step end points, respectively, to assess habitat selection using true and random movements. We used the *sf* package (Pebesma and Bivand 2023) to determine the number of linear features crossings and locations within the powerline clear cut and roads buffer polygons.

#### 2.3.4 Random Paths

**FIGURE 3:**
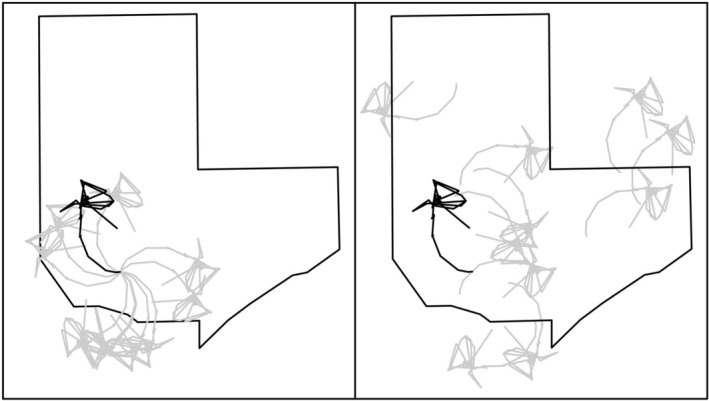
Visualization of the true starting point‐random angle (left) and random starting point‐random angle (right) random path methods within the study area. The true starting point‐random angle method keeps the path structure but randomizes the starting movement angle. The random starting point‐random angle method starts movement paths at a randomly generated location and randomizes the starting movement angle. The true movement is shown in black, while randomly generated movement paths are gray.

As a sit‐and‐wait ambush predator, *A. contortrix* movements are often punctuated with successive locations in a single location (often over days to weeks). Such movement behavior can be incompatible with correlated random walks; therefore, we utilized observed movement paths for each individual when creating randomized paths. We approached this in two ways: (1) random angles from the true starting location; and (2) random angles from random locations within the study area (Figure 3), defined as the merged polygon of the minimum convex polygon of all *A. contortrix* locations and the PV property boundaries. The combined use of these methods allowed us to assess feature crossing and habitat selection in areas that are spatially proximate to the true movement path (random angles only) and throughout the study area while replicating true paths (random starting points and angles). For the random paths analyses, we used *adehabitatHR* (Calenge 2023) in R (R Core Team 2024) to create trajectories for each animal by year. We then manipulated these trajectories by: (1) randomly changing the starting angle of each trajectory to rotate them on the starting point; and (2) randomly changing the starting point and starting angle of each trajectory to start them in different locations within the study area (Figure 3). These differing derivations of our random paths accomplish different goals. As with many other temperate snake species, *A. contortrix* annual movements and home ranges are largely dependent upon the locality of hibernacula for spring egress and fall ingress. Many of these snake species, especially *A. contortrix* , display fidelity to hibernacula (Fitch 1960; Smith et al. 2009). Therefore, the start and end of each annual movement path is tied to a fixed location. The random angle approach tests for non‐random habitat selection while keeping both the movement patterns of the individual and the starting location the same as the true movement, while the random starting point‐random angle approach tests for non‐random habitat selection with the same movement patterns of the individual but with a randomized starting location (Figure 3). For each animal's annual trajectory, we created 1000 random angle trajectories and 1000 random starting point‐random angle trajectories.

#### 2.3.5 Step Selection Function

Step selection functions (SSF) model spatially and temporally discrete decisions made by the study organism at each successive location by generating random points that are available to the organism according to their observed movement behavior. Where our random path analysis creates alternative full‐season movement paths, the SSF approach creates randomized alternative movements at each relocation. We created our SSF movement paths with the *amt* package (Signer et al. 2019) with 10 random steps for each realized step using a gamma distribution of observed step lengths and random turn angles. Groups of paired random and realized steps were given a unique identifier to ensure random steps were associated with the realized step and assigned a value of 0 or 1 denoting whether the step was random or realized, respectively.

#### 2.3.6 Context‐Dependent Movement—

The road buffer and powerline clear cut occupancy variables in our random paths and SSF analyses represent occupancy of a habitat type, rather than feature crossing. Therefore, we used a mixed effects binomial logistic regression to test for the effect of occupying road buffer habitat on the probability of movement using glmmTMB (Brooks et al. 2017). In our model, our response was a binary variable denoting whether an animal moved or remained in the same location upon each relocation. The predictor variable in this model was binary, denoting whether the animal was occupying habitat in the location prior to the move/remain response. Since we found no effect of sex in the binomial logistic portion of our step length hurdle model, we pooled all individuals in our context‐dependent movement model.

### 2.4 Data Analysis

Due to differences in the nature of the underlying data in our random paths and SSF analyses, each approach required differing statistical approaches. First, for our random paths analysis, we built distributions for each sex of the pooled number of feature crossing events and locations within the road buffer polygon for each of our approaches (random angle and random starting point‐random angle). We then calculated the percentile value (*P*_*i*_) of our true movement path within the distribution of our random paths. This approach allows us to assign a level of certainty regarding the significance of observed non‐random movement patterns, with a value of 0 signifying observed events occurring less frequently than in each of the 1000 random movement paths and a value of 1 signifying observed events occurring more frequently than in each of the 1000 random movement paths. Interpretation of these results can be considered analogous to a traditional *p*‐value approach with a value below 0.05 or above 0.95 demonstrating observed events occurred significantly less than or greater than random movement paths, respectively (Shepard et al. 2008). To test the resilience of our random paths results to individual variation, we conducted a leave‐one‐individual‐out cross validation test (LOIOCV), where *P*_*i*_ values were calculated with each individual iteratively removed to determine how much each individual affected calculated *P*_*i*_ values.

Next, we approached our SSF analysis with mixed effects conditional logistic regression using the glmmTMB package (Brooks et al. 2017). The SSF approach provides a way to alleviate concerns around spatial and temporal autocorrelation often associated with telemetry data (Thurfjell et al. 2014). Additionally, analyzing our data in a mixed effects framework allows us to control for individual variation in sample sizes and movement behavior by utilizing random effects (Muff et al. 2020). We created our conditional logistic regression model using the method described by Muff et al. (2020), where the conditional logistic regression model is reformulated as a Poisson model with a stratum‐specific random intercept (i.e., the unique identifiers described in the *Data Preparation* section). In this model, our response variable was a binary used‐random location which was predicted by our habitat selection and feature crossing variables with individual‐specific random intercepts and slopes, to account for individual variation. We calculated the relative selection strength ratio (RSS) of our predictor variables to determine the predicted strength of selection for each of our habitat selection or feature crossing variables (Avgar et al. 2017). In an SSF framework, RSS ratios estimate the selection strength of one location over another when they vary by a single variable of interest. In this application, our habitat selection and feature crossing variables are binary, therefore the predicted RSS ratios represent the odds of an *A. contortrix* crossing one of our linear features or selecting a site within 5 m of a road.

## 3 Results

We collected 1950 radiolocations from 14 (7 male and 7 female) adult *A. contortrix* from 2018 to 2023 (Appendix S1). Seven individuals were tracked over multiple years, resulting in 23 annual movement paths (12 male and 11 female; Appendix S1). The number of radiolocations per annual movement path ranged from 25 to 165, with a mean of 85 and a median of 73. The mixed effects gamma hurdle model showed no effect of sex on movement probability but did find a significant effect of sex on step length. Specifically, males displayed higher step lengths than females, with model predictions for sex‐specific step length of 21.8 m for females (95% prediction interval: 19.2–24.8 m) and 35.1 m for males (95% prediction interval: 30.9–39.8 m) (Appendix S1 Table 1).

**TABLE 1:** Parameter estimates and relative selection strength (RSS) from the step selection function (SSF) analysis for each sex.

Females						
Feature	β Parameter estimates			RSS estimates		
	Estimate	Lower 95% CI	Upper 95% CI	Estimate	Lower 95% CI	Upper 95% CI
Road crossing	−1.73	−2.50	−0.96	0.18	0.08	0.38
Trail crossing	0.58	0.22	0.95	1.79	1.24	2.59
Location within powerline clear cut	0.57	0.10	1.05	1.78	1.10	2.86
Location within 5 m of road	0.87	0.55	1.19	2.39	1.73	3.29

Males						
Feature	β Parameter estimates			RSS estimates		
	Estimate	Lower 95% CI	Upper 95% CI	Estimate	Lower 95% CI	Upper 95% CI
Road crossing	−1.52	−2.03	−1.01	0.22	0.13	0.36
Trail crossing	0.57	0.28	0.87	1.77	1.32	2.38
Location within powerline clear cut	0.55	0.12	0.98	1.73	1.13	2.66
Location within 5 m of road	0.96	0.72	1.20	2.61	2.06	3.32

### 3.1 Random Paths

**FIGURE 4:**
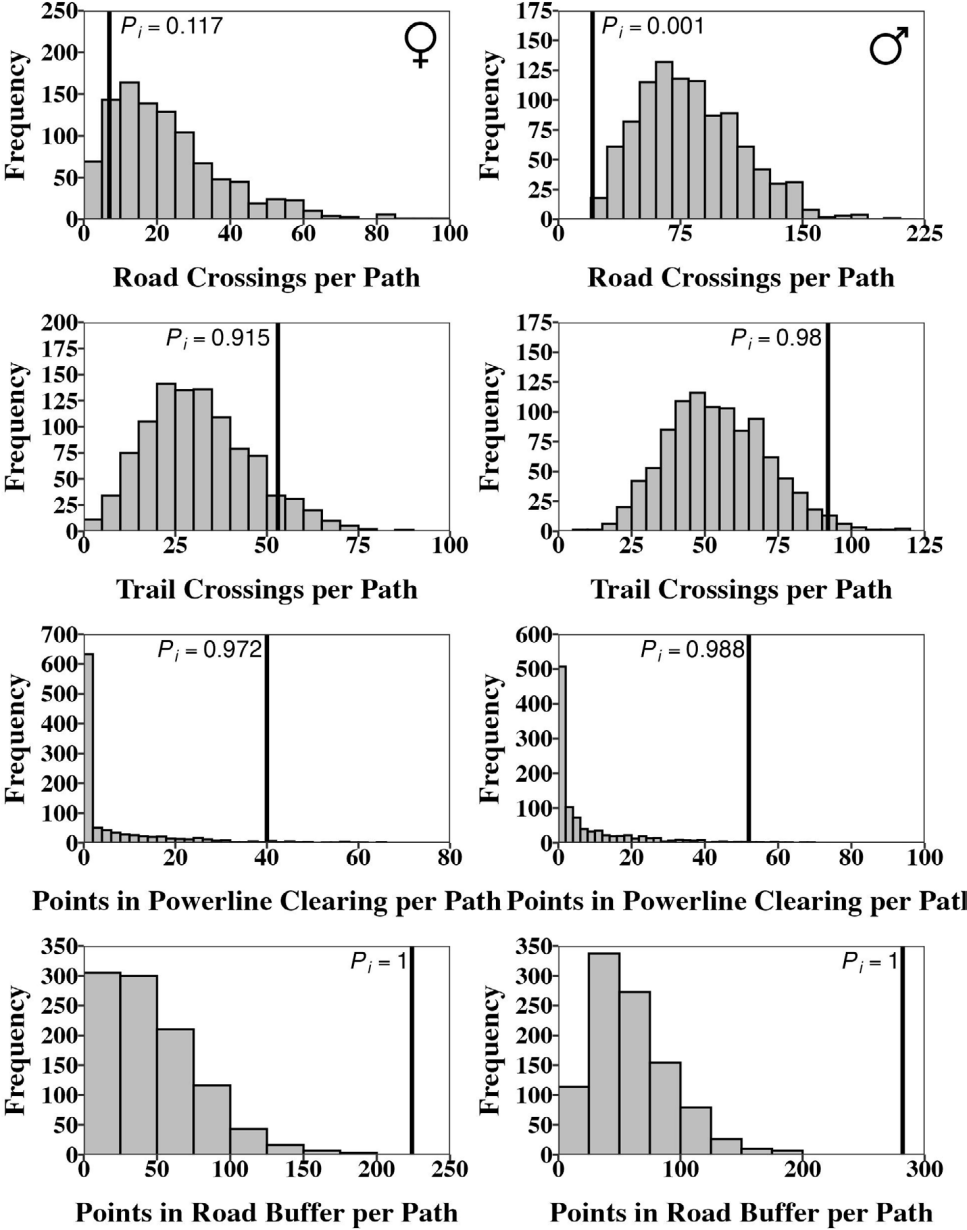
Histograms of the counts of linear features crossed by each random path generated by random starting point‐random starting angle for females (left column) and males (right column). Significance is determined by calculating the percentile of the true crossing counts within the distribution of the random crossing counts, which is shown as a black line with an associated percentile value (*P*_*i*_).

**FIGURE 5:**
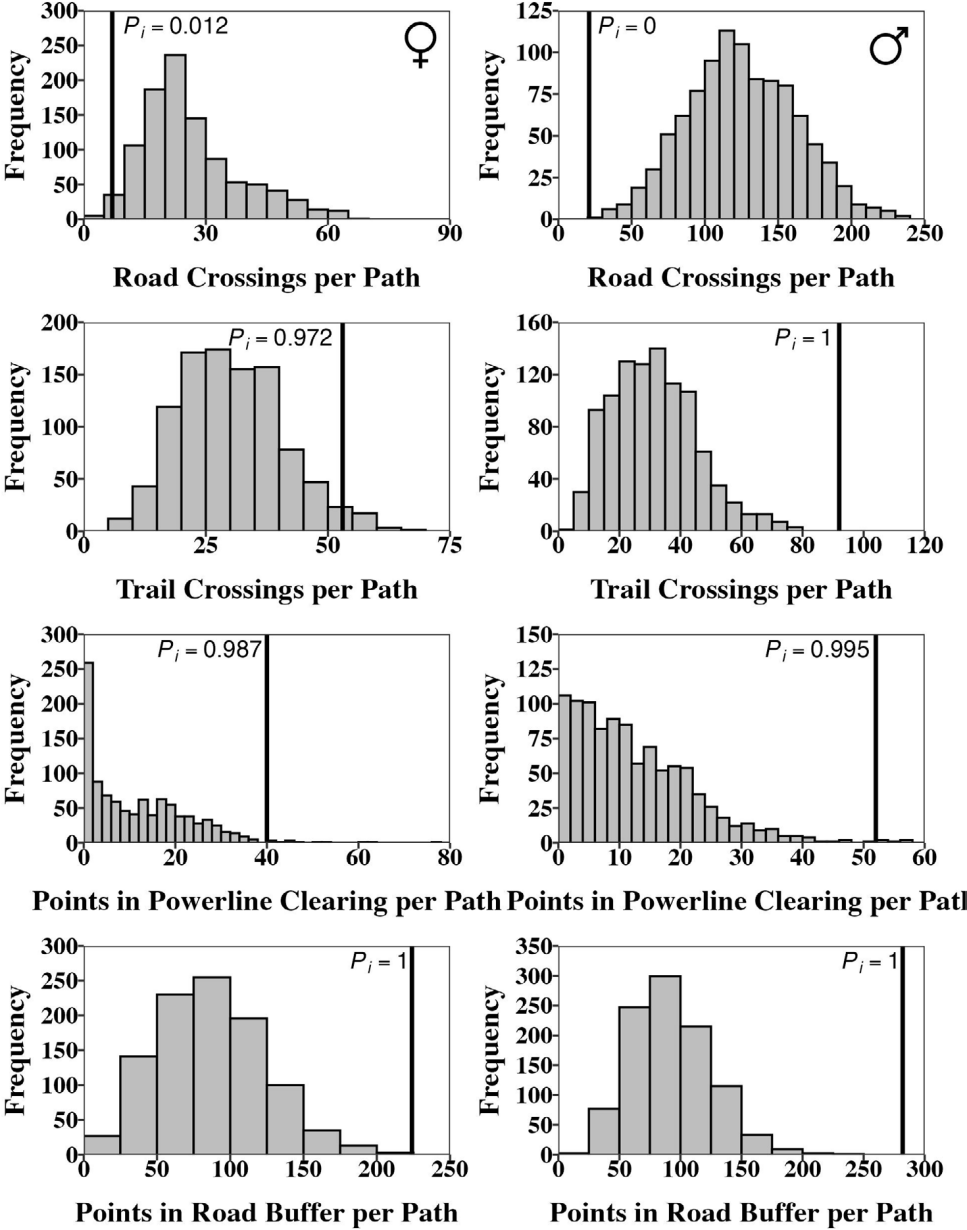
Histograms of the counts of linear features crossed by each random path generated by true starting point‐random starting angle for females (left column) and males (right column). Significance is determined by calculating the percentile of the true crossing counts within the distribution of the random crossing counts, which is shown as a black line with an associated percentile value (*P*_*i*_).

The results of our random paths analyses were generally similar for each feature type across both sexes and each of our random path generation techniques (Figures 4 and 5). Snakes of both sexes avoided crossing roads, with males crossing roads less than 100.0% (*P*_*i*_ = 0.000) and 99.9% (*P*_*i*_ = 0.001) of simulated paths in the random angle and random starting point‐random angle approaches, respectively. Females crossed roads less than 98.8% (*P*_*i*_ = 0.012) and 88.3% (*P*_*i*_ = 0.117) of simulated paths in the random angle and random starting point‐random angle approaches, respectively. Snakes of both sexes showed a preference for crossing foot trails, with males crossing more often than 100% (*P*_*i*_ = 1.000) of the random angle paths and 98.0% (*P*_*i*_ = 0.980) of the random starting point‐random angle paths, while females crossed more often than 97.2% (*P*_*i*_ = 0.972) of the random angle paths and 91.5% (*P*_*i*_ = 0.915) of the random starting point‐random angle paths. Males occupied powerline clearcuts more often than in 99.5% (*P*_*i*_ = 0.995) of the random angle paths and 98.8% (*P*_*i*_ = 0.988) of the random starting point‐random angle paths, while females crossed more often than 98.7% (*P*_*i*_ = 0.987) of the random angle paths and 97.2% (*P*_*i*_ = 0.972) of the random starting point‐random angle paths. Snakes of both sexes, evaluated with each random path method, showed a very high preference for locations within the 5 m road buffer. The observed number of locations within the buffer was higher than 100.0% of the simulated paths in all scenarios (*P*_*i*_ = 1.000).

For road crossing and 5 m road buffer occupancy, the *P*_*i*_ estimates from the LOIOCV analysis were consistently clustered at extreme values (i.e., 0 and 1), indicating that these behaviors are consistent in the population and not driven by the individuals sampled (Appendix S1). Trail crossings and powerline clear cut occupancy showed a broader range of outcomes for the LOIOCV *P*_*i*_ estimates, but the range always remained within *P*_*i*_ = 0.70–1.00, retaining the positive association with crossing these features.

### 3.2 Step Selection Function

**FIGURE 6:**
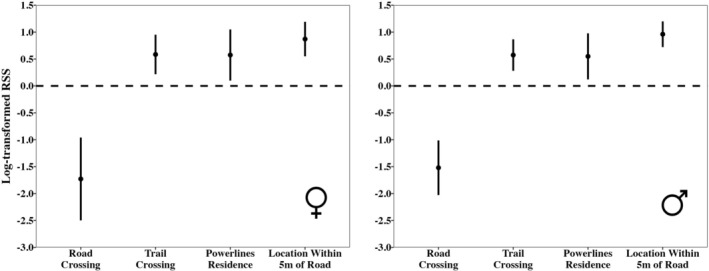
Log‐transformed relative selection strength (logRSS) [log(β_x_)] for each linear feature crossing variable in the step selection function analyses for females (left) and males (right). The predicted logRSS of each variable is signified by a black dot while the 95% confidence interval is presented as a solid black line. All logRSS predictions above zero (dashed line) indicate positive selection, predictions below zero indicate negative selection, and predictions with 95% confidence intervals that bound zero indicate no significant selection of this variable. Note that these plots show log‐transformed RSS values to improve visual interpretability of the magnitude of negative and positive selection. This transformation makes 0 the inflection point for attraction/deterrence, rather than 1 for raw RSS.

Similar to the random paths analyses, road crossings were avoided for both sexes, while trail crossings, powerline clear cuts, and locations within 5 m of a road were preferred by both sexes (Figure 6). Estimates of RSS were roughly similar between the sexes in both direction and magnitude (Table 1). Road crossing showed the greatest absolute effect on step selection, with males 4.55 and females 5.56 times more likely to avoid crossing a road than crossing it, given the choice (Table 1). Males were 1.77, 1.73, and 2.61 times more likely to select steps that crossed trails, ended in powerline clear cuts, or ended within 5 m of a road, respectively; whereas females were 1.79, 1.78, and 2.39 times more likely to select steps that crossed trails, ended in powerline clear cuts, or ended within 5 m of a road, respectively (Table 1).

### 3.3 Context‐Dependent Movement

**FIGURE 7:**
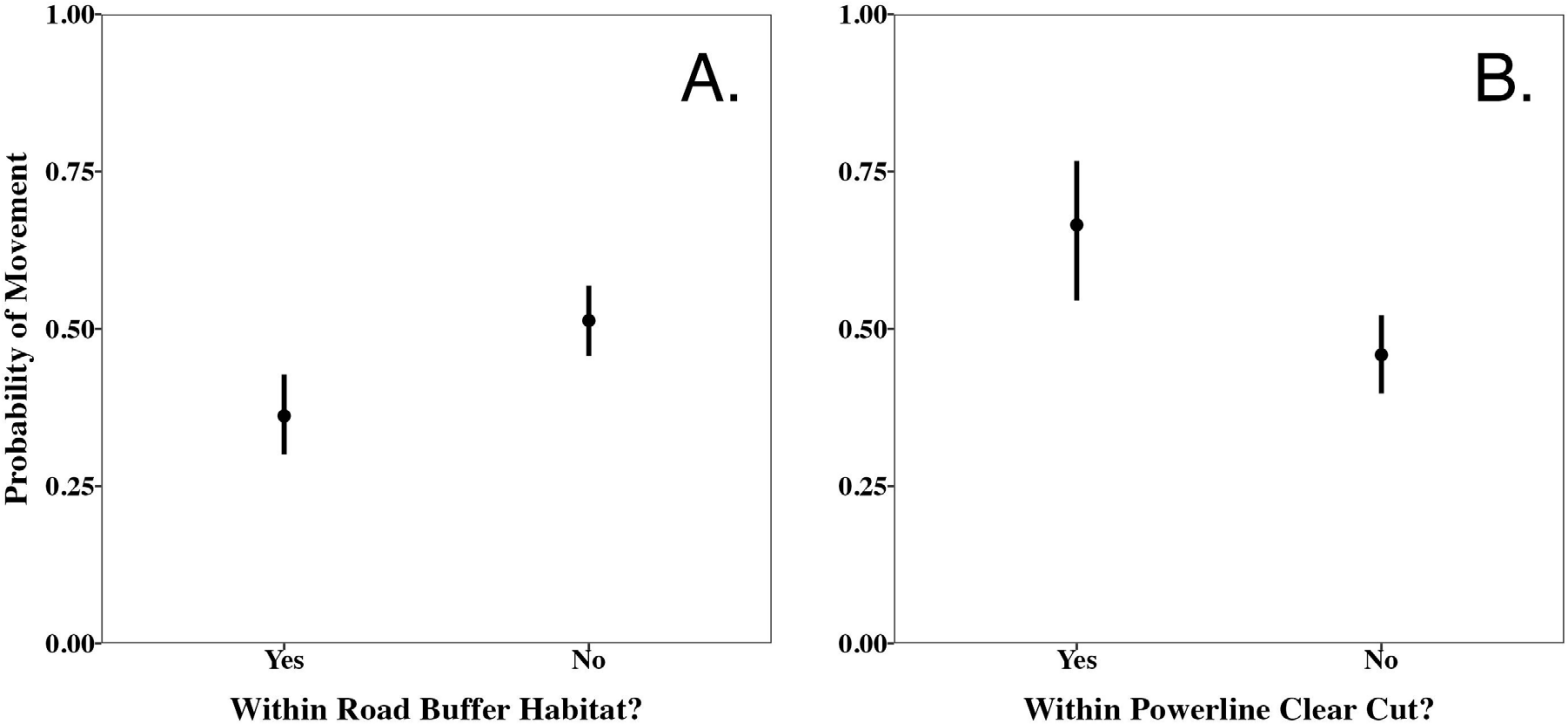
Model estimates for the mixed effects binary logistic regression testing for the probability of movement within and outside of 5 m buffer of roads (A) and within and outside of powerline clear cuts (B).

The mixed effects binary logistic regression showed that *A. contortrix* were less likely to move when they were within the 5 m buffer habitat of a road than when they were not (Figure 7A.; β = −0.61, 95% CI = −0.38 to −0.61). The probability that an individual moved when they were within the road buffer was 0.37 (95% confidence interval: 0.31–0.43) while the movement probability of movement outside the road buffer was 0.52 (95% confidence interval: 0.46–0.58). In contrast, *A. contortrix* were more likely to move when they were within a powerline clear cut than when not within one (Figure 7B.; β = 0.85, 95% CI = 0.39–1.32). The probability that an individual moved when they were within a powerline clear cut was 0.67 (95% confidence interval: 0.55–0.77) while the movement probability outside of powerline clear cuts was 0.46 (95% confidence interval: 0.40–0.52).

## 4 Discussion

Habitat fragmentation through widespread land conversion and the introduction of anthropogenic landscape features fundamentally alters the lifestyles of wildlife, often in unexpected ways. We found *A. contortrix* exhibited the strongest behavioral response in relation to roads, with animals avoiding road crossing at all scales but, counterintuitively, preferring occupancy of roadside habitat. Individuals also preferred crossing paved trails and occupying powerline clear cuts at both the season‐long movement path scale and at the level of individual step. It is clear that human‐created landscape features have a significant effect on movement in this species.

Road avoidance behavior, as seen in our study, is well‐documented in snakes and other terrestrial organisms (Andrews and Gibbons 2005; Shepard et al. 2008; Leblond et al. 2013; Peaden et al. 2017; Zeller et al. 2021). The intensity of road avoidance behavior in snakes appears to scale in proportion to more heavily trafficked roads (Robson and Blouin‐Demers 2013; Bauder et al. 2018); however, road avoidance has been repeatedly shown across taxa, road types, and adjacent habitat types (Andrews and Gibbons 2005; Shepard et al. 2008; Siers et al. 2016; Paterson et al. 2019). Despite clear road avoidance behavior in our population, roadside habitat was attractive to both sexes, evidenced by increased occupancy and higher site fidelity. An affinity for roadside habitat puts animals near roads, thus increasing the probability of road crossing through random chance. However, our SSF analysis showed the greatest absolute response was to road crossing behavior, suggesting road crossing avoidance occurs at relatively fine spatiotemporal scales.

Roads present a threat to snakes and can be a substantial source of mortality reducing population viability (Jaeger et al. 2005; Roe et al. 2006; Row et al. 2007; Fahrig and Rytwinski 2009). In addition to reducing survivorship due to roadkill events, roads affect the behavior of snakes and other terrestrial species, thus intensifying habitat fragmentation (Andrews and Gibbons 2005) and gene flow (Holderegger and Di Giulio 2010; Jackson and Fahrig 2011). Our study found roads to be a considerable barrier to movement for *A. contortrix* , a result consistent with previously studied snake populations (Andrews and Gibbons 2005; Shepard et al. 2008; Robson and Blouin‐Demers 2013; Siers et al. 2016; Bauder et al. 2018) and other terrestrial vertebrates (Laurance et al. 2004; Paterson et al. 2019; Duffett et al. 2020; Loraamm et al. 2021). Snakes were often in close proximity to a 10‐lane highway on the western edge and to a frontage road on the southern edge of the study site, but the only road crossings occurred along the local road to the nature center, a subdivision road serving three houses, and the entrance drive to the nature center proper. This shows a ranked order to road aversion by road intensity, as seen in previous studies of snakes (Robson and Blouin‐Demers 2013, Bauder et al. 2018) and other vertebrates (Tremblay and St. Clair 2009; Husby and Husby 2014; Grilo et al. 2018). Despite evidence of road avoidance in snakes across multiple studies, including this one, the underlying mechanism through which snakes perceive roads as unsuitable for crossing remains unclear. If road avoidance is not a learned behavior to avoid direct harm, snakes might avoid roads instinctively due to a lack of vegetative cover, high surface temperatures, vibrations from traffic, or other visual cues (Andrews and Gibbons 2005; Rouse et al. 2011; Paterson et al. 2019). Such “gap‐avoidance” due to a lack of cover is well‐documented in forest‐dependent birds and small mammals, for whom even narrow, low‐traffic clearings represent significant barriers to movement (Laurance et al. 2004). However, despite evidence of broader spatial scales of road avoidance behavior in other systems (Bauder et al. 2018), road avoidance in our study was limited to road crossings, with adjacent roadside behavior serving as preferred habitat for *A. contortrix* , presenting the above stated paradoxical relationship.

While roads present a formidable barrier to movement in this population, the buffer zones surrounding them create habitat for *A. contortrix* and induce site fidelity. A preference for roadside habitat has been reported in multiple taxa (Muhly et al. 2011; Rhoden et al. 2016; Peaden et al. 2017), including other members of the Viperidae (Fizzotti 2018; Sisson and Roosenburg 2023). For *A. contortrix* , roadside edge habitat, especially in areas with woody species removal, may provide high quality basking sites or access to warmer substrates for thermoregulatory purposes (George et al. 2017; Sisson and Roosenburg 2023). Indeed, in our study population, *A. contortrix* utilized the roadside habitat for gestation and overwintering refugia (B. Jellen, pers. obs.). Additionally, disturbed roadside plant communities have the potential to support higher densities of small mammal and amphibian prey species, providing greater foraging opportunities (Adams and Geis 1983; Homyack et al. 2014; Fizzotti 2018) though that was not directly examined in this study. Finally, the presence of debris, riprap, or specific vegetation types along roadsides might offer improved refuge, thermoregulatory, and/or ambush sites for a sit‐and‐wait predator, such as *A. contortrix* . Simultaneous attraction to roadside habitat and strong aversion to road crossing, suggests that individuals are sensing and avoiding roads at a fine perceptual scale. This behavior also suggests a behavioral trade‐off, where the risks of incidental road crossing while occupying roadsides must be outweighed by the benefits provided by the habitat adjacent to these features. While we did not observe any instances of road mortality in our radio‐equipped adult *A. contortrix* in this investigation, two radio‐equipped neonates and two non‐radio‐equipped adults died due to vehicular strike; therefore, the potential exists for roadside habitat to function as an ecological trap for *A. contortrix* , as observed in other systems (Ben‐Aharon et al. 2020; Hill et al. 2021; Noonan et al. 2022; Campioni et al. 2022).

Despite sex‐specific differences in step length, feature crossing and habitat selection were similar for males and females in our population. Sex‐specific differences in step length, in which males moved greater distances than females, is consistent with the movement ecology of many snake species, including other populations of *A. contortrix* (Fitch 1960; Petersen 1995; Sutton et al. 2017; Carrasco‐Harris et al. 2020). Commonly, mate searching and intrasexual competition for access to females has been cited as a source of increased male movement in pitvipers (Roth 2005; Jellen et al. 2007; DeGregorio et al. 2011; Glaudas and Rodríguez‐Robles 2011; Petersen et al. 2019; Christensen et al. 2025; Noble et al. 2025). Through random chance, increased step length in males should subject them to more road crossings; however, we found a consistently greater aversion to road crossing than for females in our random paths analyses and similar results between sexes in the SSF.

Paved foot trails crossings were consistently selected for in both the random paths and SSF analyses. The response was significant for both sexes, but the RSS was stronger in males in all analytical approaches. Paved trails in natural areas might provide similar benefits as roads (e.g., basking opportunity, improved foraging) without the threat of roadkill, which could explain the attraction to these areas without the avoidance of crossing. Additionally, there might be habitat features (such as brush and coarse woody debris) resulting from trail management immediately adjacent to the foot trails that provide attractive *A. contortrix* habitat. The presence of logs and brush along trails might contribute to the use of these features as coarse woody debris is associated with improved foraging habitat for pitvipers (Reinert et al. 2011) and is preferred habitat for *A. contortrix* (Christensen et al. 2025). Finally, locations selected for trail construction might coincide with habitat features preferred by *A. contortrix* . For example, paved foot trails, like those found at PV, are often constructed along gently sloping uplands for the ease of construction and reduced risk of flooding. Therefore, trail locations might simply coincide with suitable *A. contortrix* habitat as they are an upland specialist (Fitch 1960; Sutton et al. 2017).

Like trail crossings, powerline clear cuts were selected for across our analyses for both sexes. Because the powerline clear cuts retain natural substrate and vegetation while providing cover, unlike the paved foot trails, they may act as linear movement corridors for *A. contortrix* (Yahner et al. 2001; Bartzke et al. 2015). This result is similar to our roadside buffer analyses; however, our context‐dependent movement analysis found that snakes were more likely to move when occupying powerline clear cut habitat and less likely to move when within the roadside buffer. This suggests that, unlike roadsides, powerline clear cuts do not provide the same foraging, thermoregulation, and/or refuge benefits as the roadside habitat reinforcing their role as movement corridors, rather than resident habitat. To that end, one powerline clear cut near an overwintering refugia was heavily used by snakes during spring egress but not frequently used during the remainder of the activity season.

Although our study spanned six seasons and resulted in 23 full active season movement paths, it was based on radiotelemetry data from 14 individuals. However, the number of individuals and seasons in our data set is comparable to other radiotelemetry studies of *A. contortrix* and our number of relocations (*n* = 1950) is robust for this type of study (Sutton et al. 2017; Christensen et al. 2025). Nonetheless, given our sample size, one may argue that these data could be susceptible to outsized impacts of individual variability and issues with statistical power. Our leave‐one‐individual‐out cross validation analyses and mixed effects SSF with individual as a random effect address these issues and show minimal issues with the power of our data set to answer our study objectives. Additionally, our relocation schedule might have prevented us from detecting finer‐scale movements or brief crossing behavior. However, our relocation frequency was relatively high with a median duration between relocations of 1.05 days (IQR: 0.91–1.32 days) which is likely frequent enough to document the spatial ecology of *A. contortrix* which regularly utilizes the same location for multiple days.

## 5 Conclusions

Humans have fundamentally altered natural areas throughout the world in ways which fundamentally change how animals interact with their surrounding habitat. These effects are especially stark in urban and suburban areas, such as our study site. Our population's avoidance of road crossing reinforces the negative effects of roads on animal movement patterns seen in countless taxa and a variety of ecosystems (e.g., Andrews and Gibbons 2005; Shepard et al. 2008; Zeller et al. 2021; Leblond et al. 2013; Peaden et al. 2017). Yet, this study also found positive associations with anthropogenic features, such as powerline clear cuts and roadside habitat, which presumably create foraging, gestating, and overwintering habitat as well as movement corridors. Yet, what remains unknown is whether the anthropogenic features which seemingly induce positive associations for *A. contortrix* truly benefit this species. Our study population, and similar populations which occupy urban and suburban parks worldwide, is relegated to a relatively small natural area surrounded by an inhospitable, human‐dominated matrix. Are walking trails, powerline clear cuts, and roadside habitats truly preferred habitat for *A. contortrix* , or are they lower quality substitutes for habitat features that would naturally occur in a larger, heterogeneous landscape? And more importantly, are such human‐altered habitats capable of supporting populations of *A. contortrix* in isolated pockets within these fragmented anthropogenic landscapes? Comparative movement and habitat selection studies of *A. contortrix*, and other species, spanning urban to rural gradients are helpful to answer such questions and will become increasingly useful to understanding the implications of inevitable anthropogenic habitat alteration. Additionally, investigations on the potential implications of roads on gene flow and downstream effects, such as inbreeding, are of value to identify less obvious implications of road avoidance. Further, future studies should examine population‐level effects of anthropogenic features to determine whether anthropogenic features utilized by *A. contortrix* and other species positively affect population growth and stability.

## Author Contributions

**Ethan J. Kessler:** conceptualization (equal), data curation (supporting), formal analysis (lead), investigation (equal), methodology (equal), software (lead), validation (equal), visualization (lead), writing – original draft (lead), writing – review and editing (equal). **Shelly N. Colatskie:** conceptualization (supporting), investigation (supporting), methodology (supporting), project administration (supporting), resources (supporting), writing – review and editing (supporting). **Brittany I. Neier:** conceptualization (supporting), investigation (supporting), methodology (supporting), project administration (supporting), resources (supporting), writing – review and editing (supporting). **Benjamin C. Jellen:** conceptualization (equal), data curation (lead), formal analysis (supporting), funding acquisition (lead), investigation (equal), methodology (equal), project administration (equal), resources (equal), writing – original draft (equal), writing – review and editing (equal).

## Funding

Partial funding was provided through the St. Louis College of Pharmacy's Faculty Research Incentive Fund (award #s 116 & 4820).

## Conflicts of Interest

The authors declare no conflicts of interest.

## Supporting information


**Appendix S1:** ece373471‐sup‐0001‐AppendixS1.zip.

## Data Availability

Data and code can be accessed via the Illinois Data Bank (https://doi.org/10.13012/B2IDB‐1534499_V1). Two sets of R code have been provided to ensure both sensitive snake locations remain private and underlying data and code are available for review. One set of R code (code/acon_movement_actual.R) analyzes preprocessed data to support the conclusions presented in this manuscript. The other set of R code (code/acon_movement_rand.R) generates random snake movement paths to present the full processing pathway for the analysis of movement and feature crossing presented in this manuscript.
