# Supplementary material for: Between Risk and Refuge: Anthropogenic Linear Features Serve as Barriers, Corridors, and Habitat for Eastern Copperheads (Agkistrodon contortrix)
Source: Ecol Evol. 2026 Apr 13;16(4):e73471. doi: 10.1002/ece3.73471 (PMC13076355; doi:10.1002/ece3.73471)
Supplement: Supplementary file 1 — Appendix S1: ece373471‐sup‐0001‐AppendixS1.zip. [file ECE3-16-e73471-s001.zip › Appendix.docx]

Appendix I

**Table 1.** Parameter estimates for hurdle model of sex-specific movement probability (logit model) and step length (gamma model). Bolded parameters have 95% confidence intervals that do not span zero and indicate significance.

| *Gamma model* | *Estimate* | *Standard Error* | *Lower 95% CI* | *Upper 95% CI* |
| --- | --- | --- | --- | --- |
| **Intercept** | **3.07** | **0.07** | **2.93** | **3.21** |
| **Sex** | **0.48** | **0.10** | **0.29** | **0.68** |
|  |  |  |  |  |
|  |  |  |  |  |
| *Logit model* | *Estimate* | *Standard Error* | *Lower 95% CI* | *Upper 95% CI* |
| Intercept | 0.25 | 0.16 | -0.05 | 0.56 |
| Sex | -0.24 | 0.22 | -0.67 | 0.19 |

Appendix II

**Table 1.** Summary of the snakes monitored in this study, including their snout-vent-length (SVL) and mass at first capture, years tracked, and numbers of relocations within each year.

| ID | Sex | SVL (cm) | Mass (g) | Year | Relocations |
| --- | --- | --- | --- | --- | --- |
| 01 | Male | 69.9 | 220 | 2018 | 34 |
|  |  |  |  | 2019 | 159 |
|  |  |  |  | 2020 | 105 |
| 02 | Male | 56.8 | 180 | 2018 | 25 |
|  |  |  |  | 2019 | 154 |
|  |  |  |  | 2020 | 53 |
| 03 | Female | 58.3 | 177 | 2018 | 25 |
|  |  |  |  | 2019 | 83 |
| 09 | Female | 47.3 | 115 | 2019 | 27 |
| 10 | Female | 48.4 | 135 | 2020 | 132 |
| 13 | Female | 59.0 | 165 | 2020 | 103 |
|  |  |  |  | 2021 | 66 |
| 14 | Male | 46.5 | 95 | 2020 | 52 |
| 22 | Male | 71.9 | 280 | 2022 | 47 |
| 23 | Female | 45.7 | 75 | 2020 | 27 |
|  |  |  |  | 2021 | 73 |
| 27 | Male | 51.5 | 120 | 2021 | 165 |
|  |  |  |  | 2022 | 102 |
| 28 | Female | 46.5 | 101 | 2021 | 125 |
| 29 | Male | 54.6 | 105 | 2021 | 146 |
| 35 | Female | 60.2 | 240 | 2022 | 159 |
|  |  |  |  | 2023 | 30 |
| 39 | Male | 50.5 | 100 | 2022 | 58 |

**Table 2.** Summary of male radiolocation data, including the ID, year of the active tracking season, number of foot trail crossings, number of road crossings, number of locations within powerline clearcut, number of locations within 5m of a road, and the total number of locations for that individual in that year.

| **ID** | **Year** | **Trail Crossings** | **Road Crossings** | **Locations within Powerline Clearcuts** | **Locations within 5m of Road** | **Total Locations** |
| --- | --- | --- | --- | --- | --- | --- |
| 01 | 2018 | 6 | 0 | 0 | 7 | 34 |
| 02 | 2018 | 2 | 1 | 0 | 4 | 25 |
| 01 | 2019 | 20 | 3 | 0 | 62 | 159 |
| 02 | 2019 | 22 | 15 | 0 | 69 | 154 |
| 01 | 2020 | 15 | 0 | 0 | 25 | 105 |
| 02 | 2020 | 0 | 0 | 0 | 37 | 53 |
| 14 | 2020 | 1 | 1 | 0 | 2 | 52 |
| 22 | 2020 | 4 | 1 | 14 | 28 | 47 |
| 27 | 2021 | 11 | 0 | 9 | 7 | 165 |
| 29 | 2021 | 9 | 0 | 16 | 7 | 146 |
| 27 | 2022 | 2 | 0 | 2 | 6 | 102 |
| 39 | 2022 | 0 | 0 | 11 | 28 | 58 |

**Table 3.** Summary of female radiolocation data, including the ID, year of the active tracking season, number of foot trail crossings, number of road crossings, number of locations within powerline clearcut, number of locations within 5m of a road, and the total number of locations for that individual in that year.

| **ID** | **Year** | **Trail Crossings** | **Road Crossings** | **Locations within Powerline Clearcuts** | **Locations within 5m of Road** | **Total Locations** |
| --- | --- | --- | --- | --- | --- | --- |
| 03 | 2018 | 0 | 4 | 0 | 21 | 25 |
| 03 | 2019 | 14 | 1 | 0 | 5 | 83 |
| 09 | 2019 | 2 | 0 | 0 | 1 | 27 |
| 10 | 2020 | 21 | 1 | 0 | 57 | 132 |
| 13 | 2020 | 5 | 0 | 0 | 31 | 103 |
| 23 | 2020 | 0 | 0 | 0 | 0 | 27 |
| 13 | 2021 | 0 | 0 | 9 | 0 | 66 |
| 23 | 2021 | 0 | 1 | 1 | 22 | 73 |
| 28 | 2021 | 11 | 0 | 0 | 84 | 125 |
| 35 | 2022 | 0 | 0 | 30 | 3 | 159 |
| 35 | 2023 | 0 | 0 | 0 | 0 | 30 |

Appendix III

**Figure 1**. Leave one individual out cross-validation (LOIOCV) for the male-only random angle-only random paths analysis. Each point represents the resulting percentile (*P_i_*) of the true paths crossings or locations within 5m road buffer for data sets with each individual removed iteratively.

**Figure 2**. Leave one individual out cross-validation (LOIOCV) for the female-only random angle-random point random paths analysis. Each point represents the resulting percentile (*P_i_*) of the true paths crossings or locations within 5m road buffer for data sets with each individual removed iteratively.

**Figure 3**. Leave one individual out cross-validation (LOIOCV) for the male-only random point-random angle random paths analysis. Each point represents the resulting percentile (*P_i_*) of the true paths crossings or locations within 5m road buffer for data sets with each individual removed iteratively.

**Figure 4.** Leave one individual out cross-validation (LOIOCV) for the female-only random point-random angle random paths analysis. Each point represents the resulting percentile (*P_i_*) of the true paths crossings or locations within 5m road buffer for data sets with each individual removed iteratively.
