# Supplementary figures and images for: Between Risk and Refuge: Anthropogenic Linear Features Serve as Barriers, Corridors, and Habitat for Eastern Copperheads (Agkistrodon contortrix)
Source: Ecol Evol. 2026 Apr 13;16(4):e73471. doi: 10.1002/ece3.73471 (PMC13076355; doi:10.1002/ece3.73471)

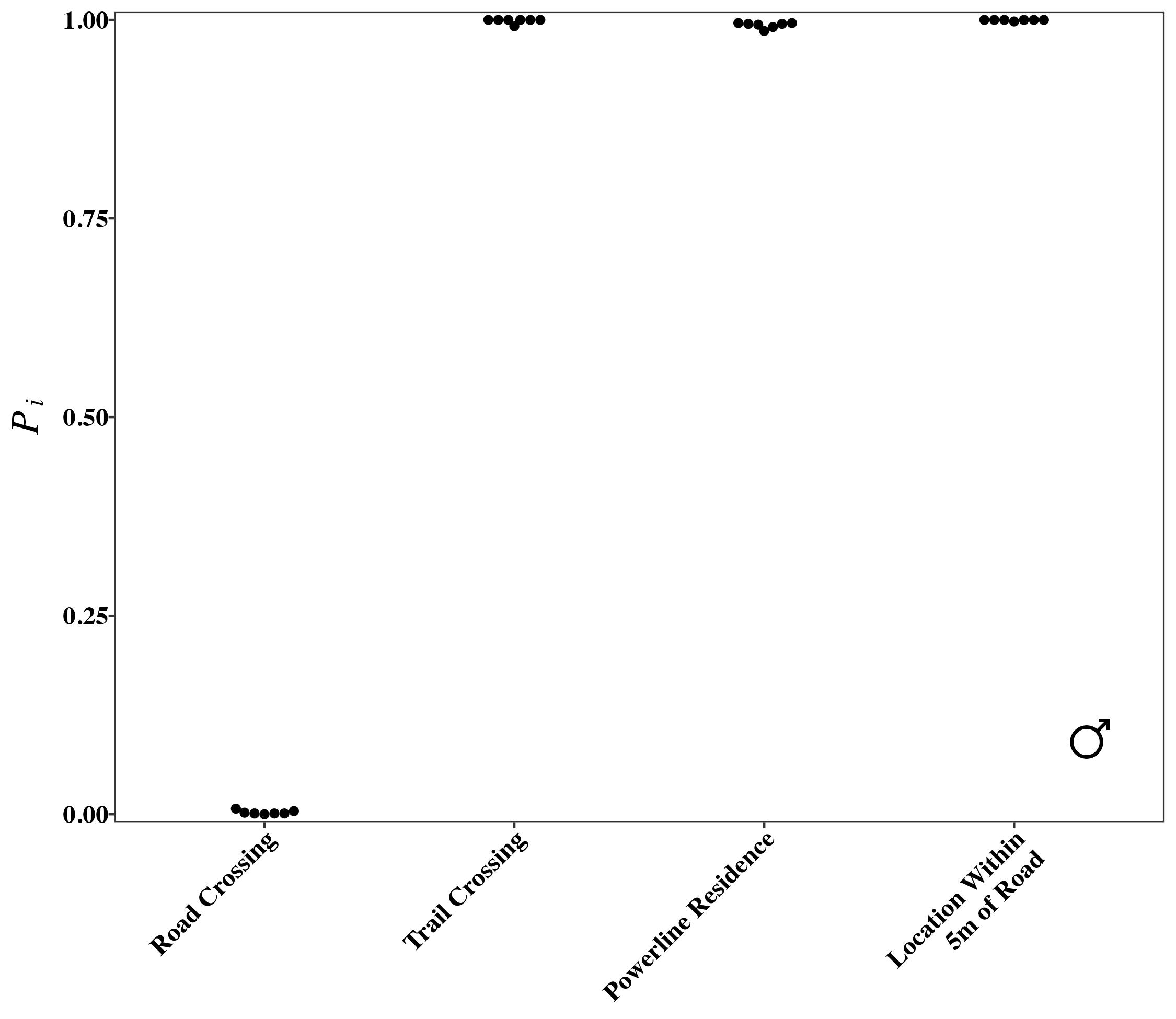

Supplement: Supplementary file 1 — Appendix S1: ece373471‐sup‐0001‐AppendixS1.zip. [file ECE3-16-e73471-s001.zip › AppIII_Fig1_LOOCV.angle.male.plot.jpg]

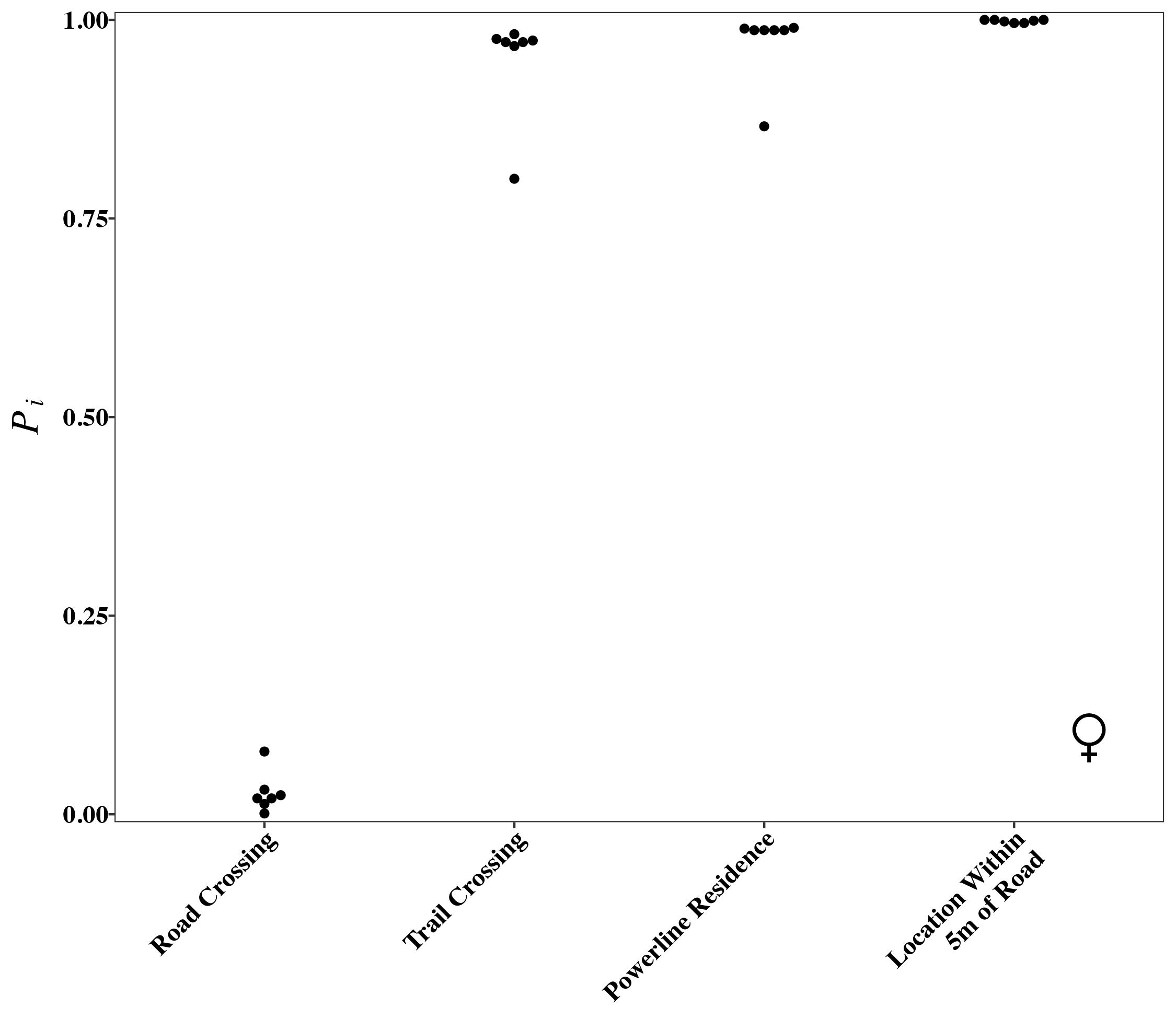

Supplement: Supplementary file 1 — Appendix S1: ece373471‐sup‐0001‐AppendixS1.zip. [file ECE3-16-e73471-s001.zip › AppIII_Fig2_LOOCV.angle.female.plot.jpg]

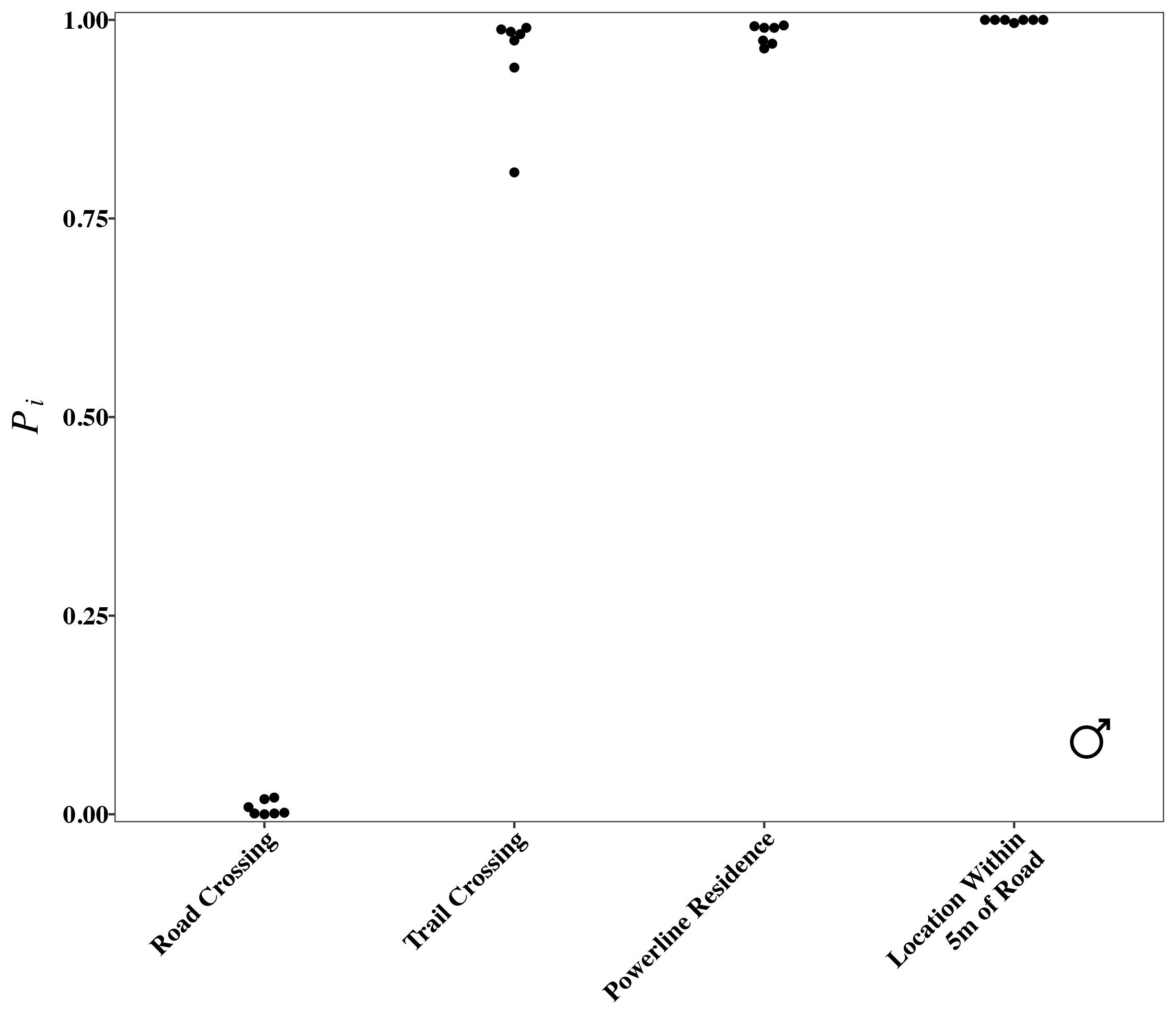

Supplement: Supplementary file 1 — Appendix S1: ece373471‐sup‐0001‐AppendixS1.zip. [file ECE3-16-e73471-s001.zip › AppIII_Fig3_LOOCV.point.male.plot.jpg]

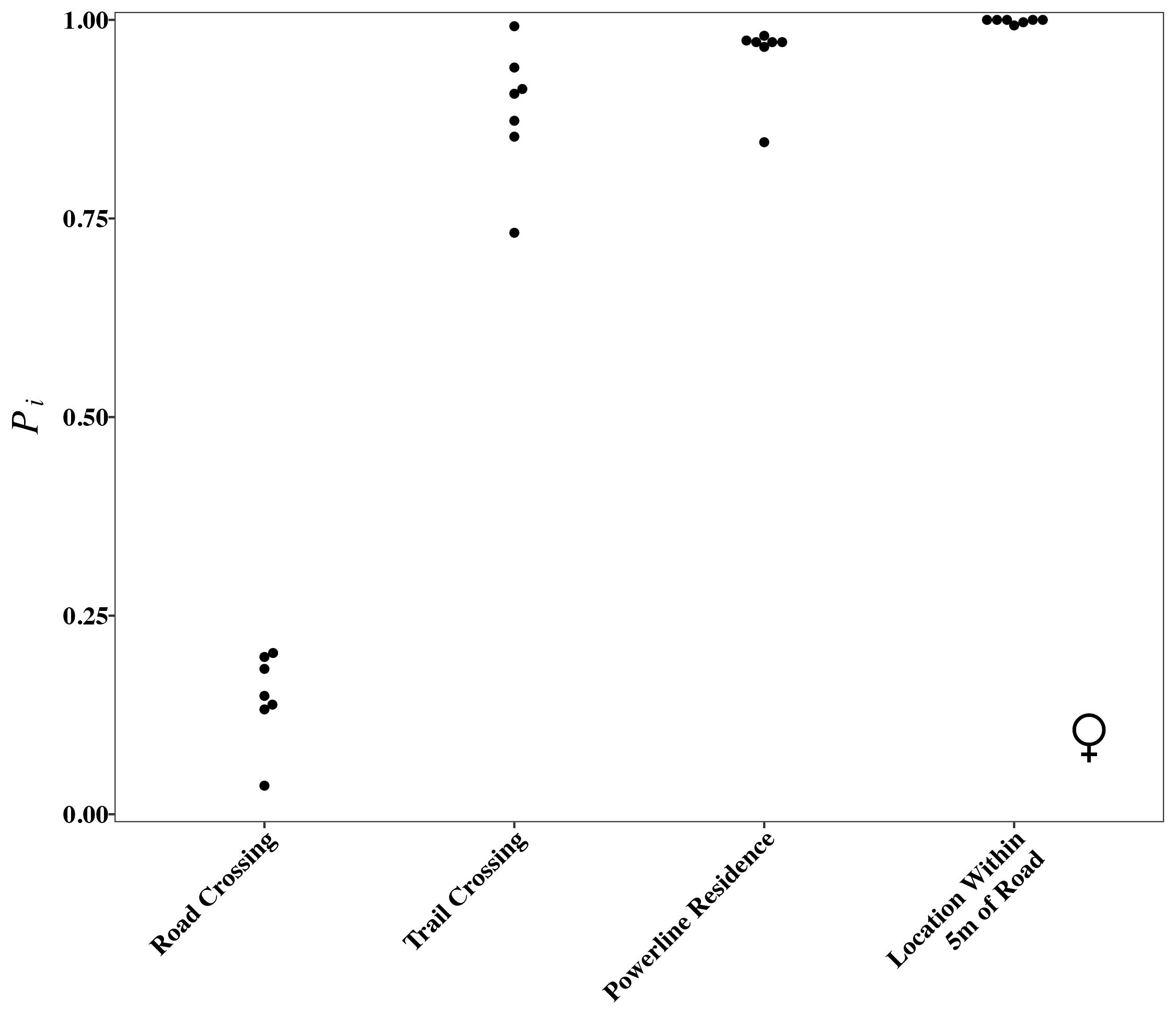

Supplement: Supplementary file 1 — Appendix S1: ece373471‐sup‐0001‐AppendixS1.zip. [file ECE3-16-e73471-s001.zip › AppIII_Fig4_LOOCV.point.female.plot.jpg]
